# Electrophysiological and Behavioral Effects of Alpha-Band Sensory Entrainment: Neural Mechanisms and Clinical Applications

**DOI:** 10.3390/biomedicines11051399

**Published:** 2023-05-08

**Authors:** Jessica Gallina, Gianluca Marsicano, Vincenzo Romei, Caterina Bertini

**Affiliations:** 1 Centre for Studies and Research in Cognitive Neuroscience, University of Bologna, Via Rasi e Spinelli 176, 47521 Cesena, Italy; jessica.gallina2@unibo.it (J.G.); gianluca.marsicano2@unibo.it (G.M.); vincenzo.romei@unibo.it (V.R.); 2 Department of Psychology, University of Bologna, Viale Berti Pichat 5, 40121 Bologna, Italy

**Keywords:** sensory entrainment, alpha oscillations, visual performance

## Abstract

Alpha-band (7–13 Hz) activity has been linked to visuo-attentional performance in healthy participants and to impaired functionality of the visual system in a variety of clinical populations including patients with acquired posterior brain lesion and neurodevelopmental and psychiatric disorders. Crucially, several studies suggested that short uni- and multi-sensory rhythmic stimulation (i.e., visual, auditory and audio-visual) administered in the alpha-band effectively induces transient changes in alpha oscillatory activity and improvements in visuo-attentional performance by synchronizing the intrinsic brain oscillations to the external stimulation (neural entrainment). The present review aims to address the current state of the art on the alpha-band sensory entrainment, outlining its potential functional effects and current limitations. Indeed, the results of the alpha-band entrainment studies are currently mixed, possibly due to the different stimulation modalities, task features and behavioral and physiological measures employed in the various paradigms. Furthermore, it is still unknown whether prolonged alpha-band sensory entrainment might lead to long-lasting effects at a neural and behavioral level. Overall, despite the limitations emerging from the current literature, alpha-band sensory entrainment may represent a promising and valuable tool, inducing functionally relevant changes in oscillatory activity, with potential rehabilitative applications in individuals characterized by impaired alpha activity.

## 1. Alpha-Band Oscillations and Neural Entrainment Mechanisms

### 1.1. Alpha-Band Oscillations Shape Visual Perception

Over the past decades, extensive research suggested that periodic fluctuations in brain activity are strongly associated with different cognitive and sensory processes and that the spontaneous brain rhythms are susceptible to functional variations, strongly affecting behavior. Among all spontaneous brain frequencies, alpha neuronal oscillations (~7–13 Hz) have been hypothesized to have a direct influence on visual processing, and its oscillatory parameters (i.e., frequency, power and phase) have been consistently associated with different aspects of visual performance [1,2,3,4,5,6,7,8,9,10,11,12,13], suggesting that the neural oscillatory activity in this frequency band may be a reliable neurophysiological hallmark of the functionality of the visual system [14]. Specifically, variations in alpha power and phase have been strongly associated with fluctuations in the excitability of the visual cortex, with the concurrent rhythmic modulation of visual performance [3,14,15,16], suggesting that neural oscillations in this frequency band actively shape visual perception modulating cortical excitability and determining the probability of perceptual reports [3,17,18]. In addition, it has been widely documented that the speed of the alpha oscillatory activity regulates the temporal resolution of the processing of visual information [16,19,20], indicating a link between alpha oscillations and the natural attitude of the visual system for processing sensory input within separate temporal windows. Overall, these extensive findings highlight the pivotal role of neuronal alpha oscillations in various aspects of visual cognition and demonstrate that modifications in alpha amplitude, phase and speed, together with changes in the perceptual performance [1,15,21], emerge during an experimental session, depending on the task requirements and cognitive state. This strongly suggests that oscillatory and perceptual activity can be considered as dynamic rather than static processes, making it possible to hypothesize that the spontaneous neuronal oscillations in the alpha frequency band can be externally modulated and facilitated.

### 1.2. Entrainment Mechanisms of Alpha-Band Neuronal Oscillations

**Figure 1:**
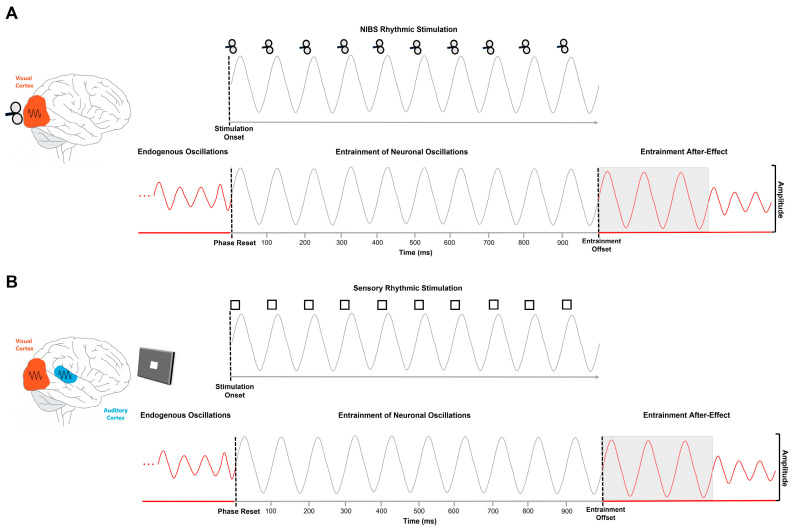
Graphical representation depicting the alpha-band neural entrainment mechanisms. (**A**) An external rhythmic force (i.e., TMS, tACS; here depicted as TMS), delivered in the frequencies of the alpha-band (here depicted as a 10 Hz entrainment), induces a phase reset of the endogenous alpha brain oscillations of the targeted visual areas, with a subsequent phase alignment, an increase in amplitude and synchronization to the external stimulation frequency. Such entrainment effects typically persist after stimulation offset for three alpha cycles (entrainment after-effect; gray shaded area). (**B**) A sensory stimulus (i.e., visual, auditory, audio-visual; here, a square, representing visual entrainment) streamed in the alpha-band (here depicted as a 10 Hz entrainment) induces a phase reset of the endogenous alpha brain oscillations of the sensory cortices, with a subsequent phase alignment, an increase in amplitude and synchronization to the external stimulation frequency. Such entrainment effects typically persist after the stimulation offset for three alpha cycles (entrainment after-effect; gray shaded area).

Based on the previously mentioned notion highlighting that oscillatory patterns can be externally modulated, a recent line of research aimed at driving functionally relevant changes in alpha oscillatory activity by directly stimulating the neural oscillating networks, through rhythmic stimulation protocols, exploiting mechanisms of neural plasticity in the visual system [22,23,24,25,26,27]. Indeed, it has been consistently documented that administering a rhythmical external force, such as Transcranial Magnetic Stimulation (TMS) e.g., [12,28,29,30], transcranial Alternating Current Stimulation (tACS) e.g., [24,31,32,33,34,35,36,37] or rhythmic sensory stimulation [15,38,39,40,41,42,43,44,45,46,47], effectively induces a temporal alignment of the endogenous brain oscillations to the external rhythmical event [29,30,48,49,50,51,52,53,54,55,56,57]. Hence, the stimulated neural substrate synchronizes its intrinsic oscillatory activity to the rhythm of the driving oscillatory force [28,44,58], typically resulting in a phase alignment [58] and power enhancement [28,39,58] of the ongoing neural oscillations, a mechanism described as neural entrainment [59] (see Figure 1). The effects of the entrainment on the ongoing oscillatory activity were demonstrated to strongly depend on different stimulation parameters e.g., [60,61]. More specifically, given the causal relationship between brain rhythms and perceptual processing, to be effective, the frequency of the entrainment should fall into a narrow bandwidth and, most specifically, the frequency of the stimulation should match the brain rhythms that are perceptually relevant for the targeted area [62]. Indeed, the mechanism of neural entrainment is based on the principle of the Arnold Tongue phenomena [60,61], which predicts that the phase synchronization between two dynamical properties of two coupled rhythmic forces occurs when the driving oscillators (i.e., stimulation frequency) approach the preferential rhythm of the targeted oscillatory system (i.e., endogenous brain oscillations). In this respect, the majority of the experimental paradigms based on entrainment protocols in vision studies showed that the periodical stimulation of parieto-occipital brain regions leads to selective and immediate changes in alpha oscillatory indices, with concurrent variations in visual performance, when the frequency of the stimulation is tuned to the preferential frequency of the visual system. Specifically, given such frequency-dependence, these studies demonstrated that a rhythmical entrainment administered at a frequency included in the alpha-band (7–13 Hz) has strong effects on both electroencephalography (EEG) activity, commonly in the posterior brain areas contralateral to the stimulation [12,28,63], and visual performance [48,64], which can last for several cycles after the entrainment offset [12,28,29,63]. In addition, it has been argued that the observed entrainment-related effects may be maximal (i.e., maximal enhancement in alpha amplitude at the stimulation offset) when the frequency of the entrainment corresponds to the participants’ individual alpha frequency (IAF; [28,31,65]). Nonetheless, it is still not completely understood whether small or large deviations from the IAF stimulation frequency may possibly result in different functional outcomes. However, a further key parameter described in the Arnold Tongue phenomena suggests that the strength of the neural entrainment outcomes might correspond to the intensity of the stimulation. In detail, it has been demonstrated that higher stimulation intensities (i.e., higher amplitude) result in a synchronization of a larger range of intrinsic frequencies around the preferential endogenous oscillatory rhythm [37,60,61,65]. Overall, these converging findings have strongly proved the efficacy of the alpha-band entrainment in driving visual performance by modulating the ongoing alpha oscillatory dynamics. Importantly, although most of the entrainment studies so far have employed non-invasive neurostimulation techniques (e.g., TMS, tACS; Figure 1, panel A), in more recent years, evidence coming from entrainment paradigms based on sensory stimulation modality has given new insights into the entrainment-related dynamics and effects on neural processing and perceptual performance. Interestingly, those studies allowed for highlighting the efficacy of the alpha-band entrainment in modulating visual abilities through intra- and cross-modal interactions between sensory systems, suggesting a potential advantage of the sensory rhythmic stimulation in inducing functionally relevant changes in neural and perceptual activity [40,41].

## 2. Physiological and Behavioral Effects of Alpha-Band Sensory Entrainment

Alpha-band unisensory (i.e., visual, auditory) and multisensory (i.e., audio-visual) entrainment has been successfully employed in a number of recent experimental investigations and was shown to be effective in inducing modulations in alpha rhythm parameters in functionally interconnected networks, both within and between the visual and the auditory systems [48,66,67,68,69,70,71], with concurrent improvements in perceptual performance [38,39,40,41,42,43,44,45,46,47,58]. Specifically, entrainment protocols are based on the principle that the first stimulus of a rhythmical train of sensory stimuli is capable of inducing a phase reset of the ongoing alpha oscillations in the visual system, synchronizing endogenous oscillatory activity to the rhythm of the external stimulation, with a consequent increase in visual detection when visual stimuli are presented in a phase relative to stimulation frequency ([38,70]; for a graphical representation of alpha-band sensory entrainment mechanisms, see Figure 1, panel B). Overall, these experimental paradigms provide strong evidence for a causal role of sensory rhythmic stimulation in enhancing neural and perceptual activity, both intra- and across-modality, and allow for clarifying how different sensory systems interact to shape perceptual performance when an external rhythmical force is administered in the range of alpha.

### 2.1. Alpha-Band Visual Entrainment

The majority of the studies probing the effects of alpha-band sensory entrainment on neuronal and perceptual activity have commonly used short trains of visual rhythmic stimulation of ~0.5–5 s ([38,39,42,43,46,58,72]; see Table 1). The short-term entrainment protocols were consistently shown to result in phase synchronization [42,43,46,58], power enhancement [38,39,42,46,58] or a shift in the frequency [44] of the ongoing alpha oscillations during the rhythmic stimulation ([65]; i.e., online entrainment), with the maximum effects being observed over the posterior scalp sites [32,39,58], supporting the idea that brain oscillating networks can be efficiently entrained when stimulated at their preferential frequency. In addition, it was shown that the entrainment-driven alpha oscillations do not return to baseline power immediately after the entrainment offset. Indeed, the alpha power exhibits a relatively high increase for approximately 2/3 consecutive alpha cycles after the stimulation ([38,39,42,46,58,65]; i.e., offline entrainment), which is frequently accompanied by an improvement in visual performance [38,39,42,43,46,58]. For instance, in a series of EEG studies, a 12 Hz visual entrainment of ~1 s in duration was effective in inducing a phase-locking and a power increase of alpha band activity in parieto-occipital scalp sites, which persisted for ~200 ms [42,58], and a clear increase in detection accuracy [42,58] and perceptual sensitivity ([15]; i.e., d’) for targets presented in-phase with the preceding stimulation in metacontrast masking paradigms. Consistent with that, in another study, a visual 10.6 Hz entrainment, administered for ~500 ms, induced a phase-locking of alpha-band oscillations in parieto-occipital areas, lasting for ~300 ms after the stimulation, and an improvement in discrimination rates for invalid trials in a cueing paradigm for in-phase visual targets [38]. In addition, in another investigation, a 10 Hz visual entrainment of 1.5 s in duration was shown to selectively induce an alpha power increase in contra-lateral early visual areas, recorded for ~300 ms after the stimulation offset, and an improvement in visual detection rates [39]. However, in contrast with the aforementioned evidence, such improvements in visual performance were observed for targets presented out-of-phase with respect to the stimulation. Therefore, the phase relationship between the driving stimulation and the subsequent target presentation has not been fully understood yet. Recently, Wiesman & Wilson [46] employed an alpha-band stimulation protocol administered at 10 Hz for 1.5 s, revealing a greater alpha event-related synchronization (ERS) and inter-trials phase coherence (ITPC) compared to a 30 Hz stimulation, persisting for ~550 ms after the offset of the stimulation. Importantly, these alpha band entrainment-induced aftereffects were accompanied by a reduced congruency effect (i.e., a smaller difference in discrimination rates between congruent and incongruent trials) in an adapted version of the arrow-based Erikson “flanker” paradigm, suggesting a robust relationship between alpha entrainment and the active inhibition of distractor stimuli. However, contrary to previous evidence, in a recent investigation, Gray and Emmanouil [73] documented no effect of ~1 s of rhythmic visual stimulation delivered at the upper (12.5 Hz) and lower (8.3 Hz) frequencies of the alpha-band on a temporal integration/segregation task of visual stimuli. Similarly, De Graaf and Duecker [72] recently documented no effect of alpha-band visual entrainment (10 Hz) on visual performance. In their web-based study, the authors employed a brief (~1 s) alpha-band visual entrainment (10 Hz), investigating whether the alpha entrainment administered on the contralateral hemifield relative to the visual target could negatively impact visual performance. Surprisingly, their results showed that alpha-band visual entrainment did not affect visual performance. However, as proposed by the authors, in the absence of M/EEG data, it is difficult to clarify whether the stimulation was effective in entraining endogenous alpha oscillations and thus in modulating visual performance.

Overall, these converging results provided evidence that short trains of visual alpha-band entrainment can induce transient, functionally relevant modulations in alpha oscillatory activity, leading to improved visual abilities and visual awareness, although some inconsistent findings suggest that the stimulation protocols, task requirements and behavioral measures used to sample visual performance can be critical for disclosing entrainment-induced effects.

### 2.2. Alpha-Band Auditory Entrainment

It has been recently hypothesized that both visual and auditory stimuli are capable of inducing a phase reset of ongoing neuronal oscillations not only within ([70]; i.e., from visual stimuli to visual areas) but also across sensory modalities ([48,71]; i.e., from auditory stimuli to the visual areas), enhancing the activity of endogenous alpha oscillations in the primary sensory visual cortices via subthreshold depolarization, synchronizing ongoing oscillatory activity across functionally interconnected nodes of sensory cortices [66,67,68,71]. Indeed, several pieces of evidence demonstrated that cross-modal phase reset (i.e., auditory to visual) of the intrinsic neuronal oscillations of the visual system results in an increase in visual detection performance and in the cyclic fluctuations of occipital alpha oscillations when visual stimuli were phase-locked to the auditory reset stimulus [48,68,70,74,75,76,77]. However, to the best of our knowledge, only three studies employed a systematic auditory alpha-band entrainment protocol with the aim of investigating its effects on performance in the visual domain ([40,41,47]; see Table 1). In detail, Ronconi and colleagues [40] investigated, in two different experiments, whether a brief (~2 s) auditory or visual entrainment administered in the pre-targets period at the alpha-band frequency (10 Hz) were able to reduce the Attentional Blink (AB) magnitude effect for reviews of AB, see: [78,79,80,81] and thus increase the accuracy detection of a second visual target (T2) presented after a first visual target (T1) in a rapid serial visual presentation (RSVP) stream. Intriguingly, their findings demonstrated that only alpha-band entrainment administered in the auditory modality—and not in the visual modality—was effective in increasing the detection of the visual target. In line with previous findings demonstrating an auditory-to-visual phase reset of endogenous alpha oscillation in the visual system [48,76,77], the auditory rhythmic stimulation facilitated target detection by synchronizing the ongoing alpha oscillations over visual cortices. Ronconi and colleagues [40] did not observe modulations of visual performance following visual stimulation. The authors proposed that auditory entrainment may be more effective in inducing stronger neuronal entrainment effects through auditory-to-visual cross-modal phase reset mechanisms of neuronal oscillations, with functional modulations of visual performance, due to the higher sensitivity of the auditory system to rhythmicity [82,83,84,85,86,87,88]. In line with this hypothesis, other evidence demonstrated that auditory rhythmic temporal sequences are capable of modulating the perception of flickering visual stimuli [89,90], taking advantage of cross-modal auditory-to-visual phase reset phenomena [66,67,68,71]. Accordingly, in a subsequent study, Ronconi and colleagues [41] confirmed the efficacy of alpha-band auditory entrainment in reducing the AB magnitude, providing EEG evidence of an association between increased posterior and frontal alpha activity during the rhythmic stream of auditory stimuli and the detection of the visual target. Furthermore, Kawashima and colleagues [47] recently employed an auditory alpha-band entrainment (10 Hz) in a web-based modality in order to replicate the findings of Ronconi and colleagues [40,41], increasing the duration of pre-target auditory entrainment (5 s) and showing that auditory rhythmic stimulation worked to increase visual detection performance in an AB task.

Overall, these converging findings underline the efficacy of the alpha-band auditory entrainment in modulating visual performance via cross-modal phase reset phenomena of alpha neuronal oscillatory activity, potentially suggesting further advantages of the auditory rhythmic stimulation in inducing entrainment, with functional consequences on visual performance [40,41].

### 2.3. Alpha-Band Audiovisual Entrainment

Intriguingly, it is conceivable that alpha-band multisensory entrainment administered through audiovisual (AV) modality may maximize the modulatory effects of the alpha neuronal oscillations with stronger consequences on various domains of the visual system, taking advantage of the cross-modal phase reset mechanisms between the functional interconnected nodes of the auditory and visual sensory systems [48,68,70,74,75,76,77]. However, to date, only two investigations have probed the modulatory efficacy of AV alpha-band entrainment regarding visual performance, employing brief (~2 s) rhythmic stimulation protocols characterized by the combination of synchronized visual and auditory stimuli ([44,45]; see Table 1). In detail, Ronconi and Melcher [44] investigated whether an AV entrainment administered in the pre-stimulus interval at the alpha (upper alpha: 11.5 Hz; lower alpha: ~8.5 Hz), theta (~6.5 Hz) and beta (~15 Hz) frequencies could shape visual temporal perception, affecting the temporal sampling of the visual system in a visual temporal integration/segregation task. Their findings demonstrated that an AV rhythmical stream delivered at the alpha band improved the temporal segregation of visual stimuli, enhancing the temporal sampling of the visual system, with respect to sensory entrainment administered at theta and beta frequencies. However, the comparison between lower and upper alpha AV entrainment revealed no significant difference in the temporal segregation/integration performance. This result appears in contrast with the previous findings highlighting that faster alpha oscillations are typically associated [91,92,93,94,95] and causally linked [96,97] with an increase in the temporal sampling of sensory systems, thus improving the temporal segregation of sensory inputs as compared to slower alpha oscillations. A potential explanation for such null result may rely on the fact that the investigators did not administer the stimulations based on the participants’ IAF, and thus, the lower and/or upper alpha stimulation frequencies were potentially set out of the participant’s IAF range, leading to an inability of the AV rhythmic stimulation to synchronize ongoing alpha oscillations via entrainment mechanisms [37,60,61,65]. However, to disambiguate this potential methodological issue, in a subsequent study, Ronconi and colleagues [45] administered an AV entrainment settled at the IAF − 2 Hz and IAF + 2 Hz of participants during the prestimulus interval in a visual temporal integration/segregation task. In line with previous evidence [91,93,96,97,98], their findings highlighted that faster and slower alpha AV stimulations accounted for opposite effects on visual temporal resolution: the AV entrainment delivered at IAF + 2 Hz improved segregation performance, whereas the IAF − 2 Hz stimulation improved integration performance. In addition, the accuracy of the segregation/integration of participants’ performance was densely sampled in time to probe whether the stimulation frequency may induce a shift of the alpha peak. Accordingly, their findings revealed a shift of the averaged alpha peak toward the stimulation frequency, highlighting faster fluctuations following IAF + 2 Hz stimulation, relative to IAF − 2 Hz.

Overall, despite empirical evidence supporting the efficacy of alpha-band AV entrainment in functionally modulating visual performance is still scarce, preliminary findings seems to support the existence of functional effects following AV entrainment [44,45], potentially opening new scenarios for the identification of optimal sensory entrainment protocols.

### 2.4. Alpha-Band Sensory Entrainment: Current State-of-the-Art and Critical Aspects

Taken together, the aforementioned evidence outlined an overall consistent effect of brief protocols of sensory entrainment, irrespective of the specific sensory modality used, in synchronizing endogenous alpha oscillatory activity over visual cortices during sensory stimulation, as well as after its offset (~three alpha cycles), with functional consequences on visual performance [38,39,42,43,46,58,99].

As mentioned, sensory entrainment effects on visual performance rely on mechanisms of phase reset of the ongoing alpha oscillations in the visual system, with a subsequent synchronization of endogenous oscillatory activity. In line with this notion, unisensory visual entrainment administered at alpha frequencies has been widely demonstrated as effective in entraining endogenous alpha oscillations of the cortical and subcortical visual pathways [28,100,101], with associated functional improvements in visual abilities and visual awareness [38,39,42,43,46,58,99]. Moreover, despite a more limited range of available empirical evidence, converging findings have recently also documented the effectiveness of auditory sensory entrainment in entraining the ongoing neuronal oscillations of the visual system, with consequent functional modulation of visual performance [40,41,47]. Indeed, it has been demonstrated that auditory stimuli are capable of inducing a phase reset of the endogenous neuronal oscillations of the visual system, synchronizing oscillatory activity through the functionally interconnected nodes between sensory systems [48,66,67,68,69,70,71]. This same mechanism of cross-modal phase reset might also subserve audio-visual entrainment-mediated effects on visual performance, although evidence of the efficacy of this sensory stimulation protocol is scarce so far [44,45].

Regarding the neural bases underlying the effects of sensory entrainment over visual areas, intra-cranial recordings in humans [102,103] and in animal models [104,105] showed that neuronal activity in both the primary visual and auditory cortex is successfully entrained in response to trains of auditory or audio-visual rhythmical stimulation, administered in a wide range of frequencies (e.g., 3–40 Hz), providing evidence for the efficacy of both unisensory auditory and cross-modal entrainment protocols in activating the neural substrate in visual areas, possibly taking advantage of direct connections between the visual and auditory cortices. In this respect, the use of tracers to highlight neural connections in monkeys uncovered the presence of direct projections of the auditory cortex to the primary visual cortex [106] and of the associative auditory cortex to the primary and secondary visual areas [107] that might subserve cortical activation in response to cross-modal rhythmic stimulation.

Overall, the existing literature strongly suggests that, although the neural circuits involved in different forms of sensory entrainment might vary as a function of the sensory modality used, the basic mechanisms orchestrating alpha-band sensory entrainment (i.e., phase reset, synchronization of endogenous alpha oscillations) are similar between sensory modalities (i.e., visual, auditory and audiovisual) of administration and have been demonstrated to be effective in modulating visual performance.

Although sensory entrainment delivered via different sensory modalities has been shown to be effective in synchronizing visual alpha neuronal oscillations and in inducing effects on visual performance, it is still unclear whether the different sensory modalities of sensory stimulation can produce different outcomes. Critically, to date, only a study conducted by Ronconi and colleagues [40] has provided a direct comparison of the potential dissociabilities of the effects on visual detection performance in a rapid serial visual presentation stream (i.e., Attentional Blink task), induced by the alpha-band sensory entrainment administered through visual and auditory modalities. A noteworthy aspect emerging from the study conducted by Ronconi and colleagues [40] is that only auditory and not visual alpha-band entrainment was effective in functionally modulating the temporal mechanisms of visual detection performance. The authors suggested that the dissociable effects of auditory and visual entrainment may potentially rely on the underlying asymmetries in sensitivity to the temporal regularities between the auditory and visual sensory systems. Accordingly, converging evidence suggests that, relative to the visual system, the auditory system is more prone to temporal regularities and thus more sensitive to rhythmic stimulation, likely due to the rhythmical nature of the auditory environment [82,83,84,85,86,87,88]. Indeed, although rhythmicity has been found to be a consistent functional property of the visual system [1,3,14,15,16,108,109,110,111], Zoefel and VanRullen [88] recently proposed that phase synchronization between endogenous oscillations and the external oscillatory rhythm is most crucial for stimulus selection in the auditory system as compared to the visual system. Such hypotheses may suggest greater entrainment modulations of the underlying neuronal oscillatory activity when the stimulation is administered in the auditory domain.

Crucially, it is conceivable that the modulatory effects of sensory entrainment may vary as a function of the sensory modality of the rhythmic sensory stream employed and, potentially, as a result of its interaction with the perceptual/cognitive processes that are investigated. Indeed, the efficacy of alpha-band visual entrainment has been mainly demonstrated in studies aiming at modulating the spatial domain of visual perceptual/attentional performance e.g., [38,39,58,99], whereas there is currently a lack of knowledge regarding its efficacy in entraining alpha oscillations in order to induce modulatory effects of the temporal mechanisms orchestrating visual performance. On the contrary, the effectiveness of alpha-band auditory entrainment has only been documented for the temporal domain of visual perception/attention [40,41,47]. Speculatively, the findings of Ronconi and colleagues [40] highlighting a modulation of visual performance only following auditory—and not visual—alpha band-entrainment seem to be in line with this perspective, suggesting a putative higher intrinsic sensitivity to the rhythmicity of the auditory system and its natural predisposition to orchestrate the temporal mechanisms of perception and cognition. Accordingly, employing an alpha-band entrainment administered via visual modality, Gray and Emmanouil [73] documented no effect on the mechanisms of temporal integration/segregation of visual stimuli. Further studies combining psychophysiological and behavioral techniques will be required to disambiguate potential dissociable effects of the alpha-band sensory entrainment administered in the visual and auditory modalities on the neuronal oscillatory activity orchestrating various aspects of visual perception.

Recently, Ronconi and colleagues [44,45] also employed multisensory AV rhythmic stimulations, exploiting the cross-modal phase reset mechanisms between auditory and visual systems [48,68,70,71,74,75,76,77]. As previously described, their findings highlighted the effectiveness of AV entrainment in entraining the ongoing alpha oscillatory activity, with functional consequences on temporal aspects of visual performance [44,45]. However, whether this modulatory effect is extendable to other domains (i.e., spatial domain) of visual perception is currently unknown, leaving to future investigations the possibility of disambiguating whether the functional modulation of the temporal and spatial aspects of the mechanisms orchestrating the visual performance may depend on the entrained sensory modality.

The examined evidence seems to suggest that the optimal stimulation parameters of alpha-band sensory entrainment that are effective in modulating visual performance have not yet been identified. To address this open question, mathematical models, such as the modified version of the Jansen–Rit neural mass model ([112]; NMM), have been developed to investigate the spectro-temporal features of the entrainment as a function of the stimulation frequency and the resonance frequency and coupling strength of resonant neural circuits in biophysical models [113]. Using this mathematical approach, Otero and colleagues [112] documented that the maximum entrainment effects are achieved only when the frequency of driving stimulation is centered to the resonance frequency of the simulated neural system, and the amplitude of the stimulus-induced oscillation in a particular brain region, as a function of the stimulation frequency, is proportional to the size of the neural population tuned to the frequency of the sensory stimulus. In addition, in the simulated neural system, the propagation of the entrainment-driven effects was restricted to cortical regions sharing similar intrinsic frequencies, hence processing the same type of information. These findings bridge models of neural oscillations and empirical electrophysiology and help shed light on the mechanisms underlying neural entrainment and the use of rhythmic sensory stimulation.

Taken together, the aforementioned evidence highlights a general efficacy of alpha-band sensory entrainment in inducing functional modulations of visual performance by synchronizing the endogenous alpha oscillations, regardless of the sensory modality in which the entrainment is administered (i.e., visual, auditory and audiovisual). At the same time, the literature reviewed suggests that the functional effects of entrainment may vary according to the sensory modality employed and its correspondence with the different aspects of visual perception that are targeted by the stimulation (i.e., temporal and spatial). However, at present, the evidence comparing the potential differences between different sensory modalities of entrainment is scarce, and future investigations are needed to disambiguate such potential differences.

Critically, in order to effectively unveil the effect of the alpha-band sensory entrainment on visual performance, in future investigations, it could also be crucial to characterize the behavioral and electrophysiological measures that may best reflect the modulated perceptual and cognitive mechanisms underlying various aspects of visual performance. The majority of the studies that systematically investigated the modulatory effects of alpha band sensory entrainment on visual performance indexed its effects by analyzing the response accuracy, documenting a general increase in visual detection accuracy following alpha-band rhythmic stimulation [38,39,40,41,42,43,44,45,46,47,58,114]. Although accuracy was found to be an efficient measure in revealing the effects of entrainment on visual performance, it has been recently proposed [115] that the measures of the signal detection theory (SDT) framework i.e., (d’ and criterion) [116], depict a clearer view of the functional dissociabilities of alpha parameters (i.e., phase, power, frequency), thus reflecting different aspects of visual performance, such as the visual temporal resolution and detection accuracy, as well as prior expectations or top-down biases. Accordingly, recent findings highlighted that higher pre-stimulus alpha power may reflect the subjective confidence of visual stimuli detection, affecting the response criterion [8,12,117], whereas alpha frequency may be more related to the spatio-temporal sampling mechanisms of the visual system orchestrating perceptual accuracy and sensitivity [12,33,91,92,96,97,98]. To date, alpha-band entrainment-induced effects were assessed by analyzing perceptual sensitivity (d’) in only one investigation [15], demonstrating higher perceptual sensitivity for visual targets presented in phase with the preceding alpha-band visual stimulation (12 Hz) in a metacontrast masking paradigm. Future investigations will be required to disentangle how sensory entrainment can differently affect perceptual sensitivity, subjective confidence, prior expectations and top-down biases by modulating the underlying parameters of the alpha oscillatory activity (however, see [22] for a first enquiry causally dissociating different oscillatory parameters such as the power and frequency of alpha and confidence and sensitivity, respectively, using neurostimulation entrainment paradigms).

Furthermore, the identification of optimal behavioral indices is of considerable importance to avoiding that the potential entrainment-induced modulations might be masked by a lack of sensitivity of the behavioral measure employed. In this regard, as previously mentioned (see Section 2.1), De Graaf and Duecker [72] recently documented no effect of alpha-band visual entrainment (10 Hz) on visual performance indexing, as a proxy measure of entrainment effects, the Reaction Times (RTs). More specifically, they investigated whether an uninformative (congruent or incongruent) cue administered at the alpha rhythm in the contralateral hemifield relative to the visual target could affect RTs. We might speculate that the task requirements and cognitive load e.g., [118] might, at least in part, have contributed to the observed lack of modulatory effects on RTs of alpha-entrainment cues. Indeed, it is conceivable that attentional costs or facilitations might prevail regarding the modulation of response times, masking visual alpha-band entrainment effects. This suggests that task requirements, behavioral responses and their relative measures might affect the emergence of sensory entrainment effects and that, therefore, these parameters should be further understood and taken in careful consideration when planning entrainment-related experimental designs.

Overall, although alpha-band sensory entrainment was widely documented as effective in modulating visual performance via the synchronization of the alpha oscillatory activity over visual cortices, further investigations are required to unravel the mechanisms underlying the different sensory entrainment paradigms, thus allowing for identifying effective protocols for promoting functional effects on alpha oscillatory activity and visual performance.

## 3. Alpha-Band Sensory Entrainment: Long-Term Effects and Clinical Applications

A noteworthy aspect that has not yet been elucidated regards whether prolonged and repeated sensory entrainment paradigms may lead to longer-lasting entrainment-induced variations in alpha oscillatory parameters and perceptual outcomes. Indeed, as extensively reported in the previous chapters, the entrainment protocols that have been employed so far were typically characterized by brief trains of periodic stimulation (~0.5–5 s), leading to transient neuronal and perceptual aftereffects, briefly outlasting the entrainment offset (~100–550 ms) [38,39,42,46,58]. A pioneering investigation showed that a prolonged audio-visual entrainment (i.e., 10 Hz) administered for 8 min resulted in either an increase or decrease in alpha power, measured for ~8 min at the entrainment offset [119]. Nonetheless, whether persistent variations in alpha oscillatory activity can be consistently achieved with prolonged sensory rhythmic stimulation remains an open question. Another crucial topic that is currently debated is whether the entrainment-related effects on alpha parameters’ activity reflect focal, rather than diffused, processes. Indeed, based on the aforementioned investigations employing short-term entrainment paradigms, it has been hypothesized that the effects of the entrainment on the ongoing alpha activity are rather local and typically occur in contralateral parieto-occipital brain regions [28,38,39]. Therefore, it still needs to be elucidated whether prolonged trains of sensory entrainment, unlike short-lasting ones, may result in more diffused and long-lasting effects that may extend to ipsilateral brain regions and/or other brain areas. This might be of particular interest in light of well-documented evidence showing alterations in the posterior alpha oscillations [120,121,122], as well as dysfunctions in alpha-band activity in large-scale networks, associated with cognitive and sensory impairments ([123,124,125]; Table 2). Indeed, occipito-parietal alpha rhythm has been consistently shown to reflect the reliable neurophysiological signature of the sensory and cognitive functionality in individuals with neuropsychiatric disorders (for a review, see: [117,123,124]), such as autism spectrum disorders (ASD) [125] or schizophrenia spectrum disorders (SSD). For instance, individuals with ASD reported a reduction in alpha power [126] and aberrant alpha power modulations [127,128] linked to impairments in the perceptual suppression of irrelevant sensory information. Moreover, individuals with ASD also show alterations in long-range structural and functional alpha connectivity (rather than in a local area; [129,130]), with a prevalence of ascending connections from posterior to anterior areas, pointing to a tendency to convey more bottom-up information [117,129,131]. In the domain of SSD, instead, a consistent slowdown of IAF [130,132,133,134,135,136] and a reduction in alpha power [137,138,139,140,141,142,143] has been observed, together with pervasive dysregulation in the way alpha oscillations instantiate phasic fluctuations and a reduction in long-range alpha coherence at rest and across different cognitive tasks [136,144]. In addition, alterations in alpha rhythm were shown to predict the structural and functional integrity of the visual system in patients affected by posterior brain damage. In particular, recent investigations regarding hemianopic and neglect patients revealed that, although residual alpha activity can be observed in response to stimuli in the blind/neglected field [145,146], these clinical populations show dysfunctional neuronal oscillatory dynamics in the alpha band, characterized by a slowdown of IAF [120,147] and a decrease in the alpha power [120,122] and connectivity [121,148] in the lesioned hemisphere that were associated with impaired visual performance in the blind and in the neglected field [120,121,122,147,148,149], strengthening the notion that post-lesional variations in alpha oscillatory activity could represent a reliable biomarker of the functionality of the visual system. In light of this evidence, prolonged alpha-band sensory entrainment protocols may represent a potential effective tool for modulating the residual impaired alpha oscillatory activity, leading to enhanced visual perceptual performance by means of the plastic reorganization of the underlying oscillating networks in these patients.

In addition to this evidence related to the alteration of alpha parameters in posterior cortices, more diffused alterations in alpha power and connectivity have also been observed in other clinical populations, such as those with dementia [150,154,155,156,157,158]. In particular, patients with Mild Cognitive Impairment [156,157,158] and Alzheimer Disease (AD; [123,124,125]) were shown to exhibit a decrease in alpha power and functional connectivity and a slowdown of the IAF [151,152], which predicted the patients’ performance in a variety of cognitive domains such as working memory, executive functions and language [150]. Importantly, the exposure to repeated sessions of sensory entrainment was shown to be beneficial for the cognitive symptoms of Alzheimer disease by enhancing the ongoing oscillations in these patients [159,160].

In addition, entrainment stimulation has also been applied to clinical populations where alpha modulations might help in ameliorating clinical signs. For instance, alpha-band visual and auditory entrainment was also shown to result in a decrease in pain intensity for acute pain induced by laser stimulation in surgery settings [161,162] and in a reduction in pain intensity ratings in patients with chronic pain [157,163]. Importantly, this reduction in pain perception following alpha-band visual entrainment might be associated with an increase in alpha power in parietal, cingulate and insular cortices [163] involved in noxious stimuli perception. Based on that, it has been hypothesized that alpha sensory entrainment leads to the inhibition of these cortical areas and a subsequent reduction in pain perception [163]. In addition, given that alpha oscillations are also involved in an attentional suppression mechanism [164] and that attention is known to influence pain perception and pain is perceived as more intense when focused on [165], it has also been suggested that the entrainment-induced reduction in pain perception might be related to the suppression of attention towards the painful stimulus, which is subsequently perceived as less intense [163]. Indeed, alpha oscillations have been associated with pain processing [166,167]. Moreover, other findings suggested that alpha-band audio-visual stimulations successfully entrained the alpha oscillations in individuals with anxiety and depressive spectrum disorders, with a beneficial impact on depressive symptoms and cognitive function, possibly due to an increase in alpha power associated with diminishment in cortical activation [168].

Overall, this accumulated evidence strongly points to the potential ameliorative and rehabilitative effects of the alpha-band sensory entrainment, making it a highly valuable tool that can be crosswise employed in various clinical populations and conditions. Based on these findings, providing proof for the efficacy of the alpha-band sensory entrainment protocols in reversing impairment in alpha activity and, in general, cognitive and sensory processing, it can be hypothesized that the administration of repeated and prolonged trains of rhythmic sensory stimulations might also be highly beneficial in the presence of altered alpha-related visual processing. However, in order to fully take advantage of entrainment-driven neural dynamics to benefit cognitive functioning and sensory processing, it should be clarified what makes the entrainment persist or how the entrainment-driven neural signal propagates across neural networks when the stimulation ends. Additionally, the functional relationship between the frequency of the stimulation and the intrinsic oscillatory properties of the targeted regions and the duration of the entrainment aftereffects should be better understood. Therefore, other experiments are required to develop a better understanding regarding the type of sensory modality and the duration and frequency of the rhythmic stimulation to employ sensory entrainment as an effective therapeutic tool that promotes cognitive and sensory enhancement by increasing the ability of distributed cortical networks to coordinate and generate brain rhythms.

## Figures and Tables

**Table 1:** Report of the main electrophysiological and behavioral findings on alpha-band sensory entrainment.

Authors, Year, Journal	Entrainment Modality	Entrainment Duration	Frequency	Visual Domain/Task	Behavioral Measures	Entrainment-Induced Effects
Mathewson et al., 2010; Cognition [15]; https://doi.org/10.1016/j.cognition.2009.11.010	Unisensory: Visual	0.800 s	12 Hz	Spatial/Metacontrast Masking paradigm	Accuracy and Signal Detection Theory (d’)	Alpha Power increase and phase-locking lasting for ~200 ms. Increased perceptual sensitivity (d’) for the in-phase target.
Mathewson et al., 2012; Journal of Cognitive Neuroscience [58]; https://doi.org/10.1162/jocn_a_00288	Unisensory: Visual	0.576 s	12 Hz	Spatial/Metacontrast Masking paradigm	Accuracy	Alpha Power increase and phase-locking lasting for ~200 ms. Increased detection accuracy for the in-phase target.
De Graaf et al., 2013; Plos One [38]; https://doi.org/10.1371/journal.pone.0060035	Unisensory: Visual	0.516 s	10.6 Hz	Spatial/Cueing paradigm (Visual discrimination task)	Accuracy	Alpha phase-locking lasting for ~300 ms and improvments in discrimination rates for in-phase targets.
Spaak et al., 2014; Journal of Neuroscience [39]; https://doi.org/10.1523/JNEUROSCI.4385-13.2014	Unisensory: Visual	1.5 s	10 Hz	Spatial/Visual detectiont task	Accuracy	Alpha power increase lasting for ~300 ms and improvements in detection rates for anti-phase targets.
Kizuk & Mathewson, 2017; Journal Of Cognitive Neuroscience [42]; https://doi.org/10.1162/jocn_a_01058	Unisensory: Visual	1 s	12 Hz	Spatial/Metacontrast Masking paradigm	Accuracy	Alpha Power increase and phase-locking lasting for ~200 ms. Increased detection accuracy for the in-phase target.
Wiesman & Wilson, 2019 [46]; Journal of Cognitive Neuroscience; https://doi.org/10.1162/jocn_a_01422	Unisensory: Visual	1.5 s	10 Hz	Spatial/Adapted version of the arrow-based Erikson “flanker” paradigm	RTs	Increased alpha ERS and ITPC lasting for ~550 ms, with reduced discrimination rates for distractor stimuli.
Gray & Emmanouil, 2020; Psychophysiology [73]; https://doi.org/10.1111/psyp.13480	Unisensory: Visual	1 s	Lower Alpha: 8.3 Hz; Upper Alpha: 12.5 Hz	Temporal/Two-flash fusion task	Accuracy and psychometric thresholds	No effects of alpha-band visual entrainment on the visual temporal integration/segregation task. Increased alpha power and ITPC during and following alpha entrainment.
De Graaf & Duecker, 2022; European Journal of Neuroscience [72]; https://doi.org/10.1111/ejn.15483	Unisensory: Visual	0.717 s	10 Hz	Spatial/Cueing paradigm (Visual discrimination task)	RTs	No effects of alpha-band visual entrainment on visual detection performance (RTs).
Ronconi et al., 2016; Psych. Res. [40]; https://doi.org/10.1007/s00426-015-0691-8	Unisensory: Visual and Auditory	2 s	10 Hz	Temporal/Attentional Blink	Accuracy	Only auditory—but not visual—alpha-band entrainment improved visual target detection accuracy, reducing the AB magnitude.
Ronconi et al., 2016; NeuroReport [41]; 10.1097/WNR.0000000000000565	Unisensory: Auditory	2 s	10 Hz	Temporal/Attentional Blink	Accuracy	Increase in the alpha power during auditory entrainment, associated with improvements in the visual detection of the target.
Kawashima et al., 2022; European Journal of Neuroscience [47]; https://doi.org/10.1111/ejn.15760	Unisensory: Auditory	5 s	10 Hz	Temporal/Attentional Blink	Accuracy	Auditory alpha-band entrainment improved visual target detection accuracy, reducing the AB magnitude.
Ronconi & Melcher, 2017; J. Neurosc. [44]; https://doi.org/10.1523/JNEUROSCI.1704-17.2017	Multisensory: Audiovisual	2 s	Lower Alpha: ~8.5 Hz; Upper Alpha: ~12 Hz	Temporal/Integration and Segregation task	Accuracy	Phase alignment of the perceptual oscillation, with two different power spectra that peaked toward the entrainment stimulation frequency.
Ronconi et al., 2018; Scientific Reports [45]; https://doi.org/10.1038/s41598-018-29671-5	Multisensory: Audiovisual	2 s	IAF − 2 Hz, IAF + 2 Hz	Temporal/Integration and Segregation task	Accuracy	IAF + 2 Hz entrainment improved the segregation performance of visual stimuli, whereas IAF − 2 Hz improved integration. Behavioral oscillations were almost in anti-phase between the two tasks: the highest integration performance corresponded to the lowest segregation performance.

**Table 2:** Summary of the alterations of the oscillatory parameters in the alpha-band that could represent a valuable target for rhythmic sensory stimulations administered in the alpha-band in different clinical populations. (AD: Alzheimer Disease; MCI: Mild Cognitive Impairment; ASD: Autism Spectrum Disorder; SSD: Schizophrenia Spectrum Disorder.)

Cognitive Impairment	Acquired Posterior Brain Lesion	Psychiatric Conditions
AD Reduced alpha functional connectivity in bilateral temporal regions at rest [150]. Slowdown of the IAF and reduced alpha power all over the scalp at rest [151]. *MCI* Slowdown of the IAF and reduced alpha power in anterior, temporal and parietal areas at rest [152].	Hemianopia Slowdown of IAF and power imbalance in favor of the intact hemisphere in parieto-occipital regions at rest [120]. Reduced alpha desynchronization in anterior, parietal and posterior areas at rest [122]. Reduced intra-hemispheric alpha connectivity and reduced alpha short-range connections in the lesioned hemisphere at rest [121]. Reduced alpha ERD and functional connectivity in the visual areas of the lesioned hemisphere during tasks [148]. *Neglect* Slowdown of the IAF in fronto-parieto-occipital regions, alpha power asymmetry in favor of the intact hemisphere in parietal areas, reduced alpha functional connectivity between lesioned fronto-parietal areas and reduced inter-hemispheric alpha connectivity in parietal areas [147].	ASD Reduced alpha power in parieto-occipital areas [127]. Altered alpha connectivity at rest in long-scale networks [127,153]. *SSD* Slowdown of the IAF [130,132,133,134,135,136] and reduced alpha power [137,138,139,140,141,142,143] in parieto-occipital areas. Reduced alpha coherence in long-scale networks at rest and during task execution [136,144].

## Data Availability

No new data were created or analyzed in this study. Data sharing is not applicable to this article.
